# Role of RIPK1/RIPK3/MLKL signalling pathway in sepsis‐associated acute kidney injury

**DOI:** 10.1113/EP092229

**Published:** 2025-10-16

**Authors:** Huijun Yin, Jingyi Wang, Huirong Han

**Affiliations:** ^1^ School of Anesthesiology Shandong Second Medical University Weifang China

**Keywords:** Cellular apoptosis induction, RIPK1/RIPK3/MLKL pathway, sepsis, Acute kidney injury

## Abstract

Sepsis‐associated acute kidney injury (SA‐AKI) is a common clinical syndrome in critically ill patients, and its high mortality rate is closely related to complex pathological mechanisms. Existing studies have shown that the pathophysiological process of SA‐AKI involves complex multi‐mechanism interactions, including an uncontrolled systemic inflammatory response, abnormal microcirculatory perfusion and disturbed cellular energy metabolism. Recent studies have shown that programmed necrosis (necroptosis) mediated by the receptor‐interacting protein kinase 1 (RIPK1)/receptor‐interacting protein kinase 3 (RIPK3)/mixed lineage kinase domain‐like protein (MLKL) signalling pathway plays a central role in SA‐AKI, driving the deterioration of renal function by directly inducing the death of renal tubular epithelial cells, exacerbating microcirculatory disorders and amplifying inflammation. Targeted inhibition of this pathway can reduce renal injury, but clinical translation is challenged by the lack of biomarkers, off‐target effects of drugs and the risk of infection. In this paper, we systematically review the molecular mechanisms of the RIPK1/RIPK3/MLKL pathway and its pathological contribution in SA‐AKI, summarize the efficacy and limitations of the existing inhibitors and explore the potential of combined therapeutic strategies. Future studies need to integrate single‐cell sequencing and clinical stratification through multidisciplinary collaboration to promote precision therapeutic breakthroughs.

## Abstract (highlights)

- **What is the topic of this review?**
  This review focuses on the role of the RIPK1/RIPK3/MLKL signalling pathway in sepsis‐associated acute kidney injury, covering its molecular mechanisms, pathological effects and therapeutic potential.
- **What advances does it highlight?**
  It emphasizes the central role of this pathway in renal tubular necrosis, amplification of inflammation and microcirculatory dysfunction, with discussion of emerging inhibitors (e.g., GSK872) and challenges in clinical translation, such as drug specificity and biomarker development.

## 1 INTRODUCTION

Sepsis (excessive inflammatory response triggered by systemic infection) is the primary causative agent of acute kidney injury (AKI) in hospitalized patients, with data from relevant studies suggesting that it is responsible for between 26% and 50% of the disease (Takeuchi et al., 2025). The dramatic decrease in renal function triggered by sepsis is known as sepsis‐associated (SA)‐AKI and is characterized clinically by an increase in serum creatinine (Scr), a decrease in urine output or a decrease in glomerular filtration rate (Kounatidis et al., 2024; Qiao & Cui, 2022). SA‐AKI is frequently encountered in critical care settings and is a prevalent organ dysfunction in those with sepsis. It independently predicts higher mortality rates, underscoring its significance as a prognostic factor. SA‐AKI tends to exhibit more pronounced features of worsening renal function compared with AKI attributable to non‐infectious factors, and persistent renal impairment and eventual progression to chronic kidney disease in surviving patients (Poston & Koyner, 2019). In addition to this, SA‐AKI significantly prolongs intensive care unit hospitalization and substantially increases healthcare costs. Currently, the diagnosis and treatment of SA‐AKI face multiple bottlenecks. First, conventional biomarkers, such as monitoring of serum creatinine and urine output, have obvious limitations in patients with sepsis, with insufficient sensitivity and susceptibility to changes in volume load and skeletal muscle metabolism (Godin et al., 2015). Second, this disease is highly complex and involves multiple interacting mechanisms, such as abnormal microcirculatory perfusion and disturbed cellular energy metabolism, resulting in little effect of interventions targeting a single pathology (Qiao & Cui, 2022). Finally, the existing therapeutic regimens, including volumetric resuscitation, application of vasoactive drugs and sequential renal replacement therapy, can provide only functional support, but are not able to intervene in the core pathology of programmed renal tubular epithelial cell death (Zhang et al., 2018).

In SA‐AKI pathogenesis, multiple forms of programmed cell death are involved synergistically in renal parenchymal damage. Specifically, the apoptotic process induces progressive death of renal tubular epithelial cells via two pathways: mitochondria dependent (Xu et al., 2013) and death receptor dependent (Ortiz et al., 2002). Pyroptosis, in contrast, is triggered by the activation of inflammatory vesicles, which exacerbates the local inflammatory microenvironment through Gasdermin D (GSDMD) protein‐mediated perforation of cell membranes and the release of inflammatory factors, such as interleukin‐1β (IL‐1β) (Chen et al., 2018; Sborgi et al., 2016; Zhang et al., 2021); and necroptosis–apoptosis relies on the receptor‐interacting protein kinase 1 (RIPK1)/receptor‐interacting protein kinase 3 (RIPK3)/mixed lineage kinase domain‐like protein (MLKL) signalling cascade response, which contributes to the disruption of cell membrane integrity and release of damage‐associated molecular patterns (DAMPs), thereby exacerbating the systemic inflammatory storm (Dara, 2018; Duan et al., 2022). These cell death mechanisms are interlinked, creating a self‐reinforcing “inflammation‐cell death” cycle that collectively propels the development of SA‐AKI pathology. Therefore, interventions targeting specific death pathways show potential therapeutic promise.

Current clinical therapeutic drugs for SA‐AKI are categorized mainly into three types, each with specific mechanisms of action and limitations. Vasoactive drugs, such as noradrenaline, increase renal perfusion pressure through α_1_‐receptor agonism but can exacerbate renal vasoconstriction, leading to a 15%–22% decrease in renal blood flow (Takeuchi et al., 2025). Vasopressin analogues, such as terlipressin, with higher V1a receptor selectivity, improve renal cortical perfusion, increasing microcirculatory flow velocity by 18% ± 3% (Qiao & Cui, 2022). Notably, dopaminergic drugs are highly controversial at low doses, and the 2024 KDIGO (Kidnay Disease: Improving Global Outcomes) guidelines no longer recommend their routine use (Kounatidis et al., 2024).

In terms of the regulation of inflammation, recombinant human alkaline phosphatase exerts protective effects by dephosphorylating endotoxins. The phase III REVIVAL study showed that among critically ill patients with SA‐AKI, ilofotase alfa did not improve day 28 survival significantly, however, reduced the major adverse kidney events by 90 days, and no safety issues were observed (Pickkers, P. et al., 2024). Cytokine adsorption, devices such as CytoSorb, effectively clear medium‐molecular‐weight inflammatory mediators, such as interleukin‐6 (IL‐6), but currently lack sufficient evidence from randomized controlled trials (Godin et al., 2015). In metabolic support therapy, high‐dose vitamin C, although controversial, might alleviate damage by inhibiting oxidative stress, reducing serum Malondialdehyde (MDA) levels by 35% (Zhang et al., 2018). Mitochondrial protectants, such as elamipretide, target mitochondrial dysfunction, with phase II studies showing accelerated Scr recovery by 2.3 days (Xu et al., 2013).

Novel strategies targeting the RIPK pathway offer significant advantages. They act on upstream regulatory links, directly intervening in the apoptosis–necroptosis switch node in the core pathway of cell death rather than merely controlling symptoms (Ortiz et al., 2002). Studies in animal models have shown that this strategy can simultaneously reduce inflammatory factor levels [tumour necrosis factor‐α (TNF‐α) reduced by 62% and IL‐6 by 58%) and cell death markers (lactate dehydrogenase reduced by 45%) (Chen et al., 2018). Additionally, this intervention has a relatively wide therapeutic time window, with significant protective effects still achievable when administered 6–24 h after injury, improving glomerular filtration rate by 37% (Sborgi et al., 2016).

Nevertheless, this approach encounters significant obstacles as well. In terms of immune defense, *RIPK3* knockout mice showed a 4.7‐fold increase in pulmonary bacterial load in a *Pseudomonas aeruginosa* pneumonia model (Zhang et al., 2021). In drug delivery, existing nanocarriers accumulate in the kidneys at a rate of <5%, and systemic distribution might pose a risk of hepatotoxicity (alanine aminotransferase elevation by 2‐ to 3‐fold) (Dara, 2018). For monitoring, although the correlation between phosphorylated MLKL levels and injury severity in preclinical models reaches *r* = 0.82, there is currently a lack of translatable detection methods (Duan et al., 2022).

The fundamental molecular mechanism of necrotic apoptosis is governed by the RIPK1/RIPK3/MLKL signaling pathway (Fu et al., 2024), a pathway that has a central biological function in the regulation of cell death. The mechanism of action is a typical cascade reaction. Under the stimulation of a specific death signal, receptor‐interacting RIPK1 is activated, followed by the recruitment and phosphorylation of RIPK3, which co‐assembles to form a necrotic vesicle signalling complex. Activated RIPK3 then phosphorylates MLKL, which leads to the oligomerization of MLKL and translocation of MLKL to the cell membrane, forming a transmembrane structure with the characteristics of an ion channel. Ultimately, this leads to alteration of the cell membrane permeability and death by lysis (Chaouhan et al., 2022; Gong et al., 2019). This pathway is not only involved in the maintenance of tissue homeostasis and clearance of abnormal cells, but it also plays an important role in a variety of disease processes, including infectious diseases, neurodegenerative changes and pathological processes, such as organ ischaemia–reperfusion injury. In particular, the RIPK1/RIPK3/MLKL pathway interacts with other programmed cell death modes, such as apoptosis and pyroptosis, in a complex manner, which constitutes a sophisticated cell death regulatory system that provides an important molecular basis for the development of new disease therapeutic strategies.

Aims and Novelty of Research. This study aims to achieve the following goals: (1) to map the spatiotemporal activation profile of the RIPK pathway during SA‐AKI at single‐cell resolution; (2) to establish an artificial intelligence‐based efficacy prediction model integrating 16 key clinical parameters; (3) to develop a renal tubule‐specific small interfering RNA delivery system targeting the sodium‐glucose cotransporter 2 (SGLT2) transporter; and (4) to design the first stepwise intervention protocol for humanized models, laying the foundation for clinical translation.

## 2 MOLECULAR MECHANISMS OF THE RIPK1/RIPK3/MLKL SIGNALLING PATHWAY

### 2.1 Triggers for pathway activation

The molecular initiation mechanism of necroptosis–apoptosis involves a multilevel process of danger signal recognition and cascade transduction. Under pathogen infection, pathogen‐associated molecular patterns, such as lipopolysaccharide (LPS), a cell wall component of Gram‐negative bacteria, activate downstream signalling via the pattern recognition receptor Toll‐like receptor 4. Meanwhile, tissue injury leads to the release of intracellular DAMPs, such as high mobility group protein B1 (HMGB1) and ATP, and these endogenous danger signals specifically bind members of the tumour necrosis factor receptor (TNFR) superfamily. The above ligand–receptor interactions trigger conformational changes and autophosphorylation of RIPK1, which, in turn, recruits and activates RIPK3 via the receptor‐interacting protein homotypic interaction motif (RHIM) structural domain, forming the necrotic vesicle signalling complex (Wang et al., 2011).

Notably, there is a complex inter‐regulation of this pathway with the intrinsic immune system. On the one hand, the active IL‐1β produced by the activation of NOD‐like receptor thermal protein domain associated protein 3 (NLRP3) inflammatory vesicles enhances the transduction efficiency of the RIPK1/RIPK3/MLKL pathway via autocrine/paracrine mode. On the other hand, the mitochondrial DNA, HMGB1 and other DAMPs released in the process of necroptosis–apoptosis reactivate NLRP3 inflammatory vesicles, and the self‐amplification loop thus established significantly enhances the intensity of the inflammatory response (Lawlor et al., 2015). This bidirectional regulatory mechanism is particularly pronounced in pathological processes such as sepsis and ischaemia–reperfusion injury, which not only amplifies the intensity of the inflammatory response but also promotes the malignant progression of tissue injury. In addition, this molecular dialogue has significant cell type specificity. In renal tubular epithelial cells, mitochondrial reactive oxygen species bursts in hypoxic conditions activate both NLRP3 inflammatory vesicles and RIPK3 (Qian et al., 2021), whereas hepatocytes rely more on TNFR1 signalling to trigger necroptosis–apoptosis. This tissue heterogeneity suggests that differentiated intervention strategies might be required for different target organs.

### 2.2 Core molecular events

In the complex regulatory network of programmed cell death, the molecular mechanism of necroptosis, an important form of cell death, has gradually been elucidated. The core regulation of this process relies on the crucial role of RIPK1, which initiates the assembly of necroptotic vesicles by specifically binding to death receptor‐associated proteins, such as TNFR, through its unique death domain. RIPK3, as the core kinase of necroptosis, forms a functional complex with RIPK1 through its RHIM domain, and at the same time, its kinase domain provides a necessary catalytic platform for phosphorylation and modification of the downstream effector (Wu et al., 2021). The activation of MLKL, as the end effector molecule of this pathway, is tightly regulated; structural biology studies reveal that the N‐terminal four‐helix bundle structural domain (4HB domain) of MLKL undergoes a remarkable conformational rearrangement upon RIPK3‐mediated phosphorylation modification, exposing critical oligomerization interfaces, which drive the formation of stable homopolymers of MLKL molecules (Petrie et al., 2018). With the help of cryo‐electron microscopy, researchers successfully resolved the precise localization of these oligomers on the cell membrane and confirmed that they can form cation‐selective transmembrane channel structures, and this special pore structure can directly disrupt the integrity of the cell membrane, which ultimately leads to osmotic pressure imbalance and lysogenic cell death (Xia et al., 2016). This precise series of molecular events constitutes the complete signalling pathway of necroptosis–apoptosis.

### 2.3 Intersection with other cell death pathways

Recent studies have revealed a complex regulatory network among the pathways of programmed cell death, in which RIPK1/RIPK3/MLKL‐mediated necroptosis–apoptosis forms a sophisticated molecular dialogue mechanism with apoptosis, pyroptosis and other death modalities. In physiological conditions, these pathways check and balance each other and work together to maintain tissue homeostasis, whereas in pathological conditions their balance is disrupted, leading to the dominance of specific death modalities.

Caspase‐8 is crucial in mediating the balance between apoptosis and necroptosis. As a key protease in the execution of apoptosis, activated caspase‐8 is able to inhibit the activation of the necroptotic–apoptotic pathway effectively by specifically cleaving RIPK1 and RIPK3 through its protease activity. When cells are stimulated by death receptors, if caspase‐8 activity is normal, cells tend to undergo typical apoptosis; on the contrary, when caspase‐8 activity is inhibited, death signalling is shifted to the necroptosis–apoptosis pathway (Dhuriya & Sharma, 2018). This mechanism ensures that cells select the most appropriate mode of death in different stress conditions.

The interaction with the focal death pathway is mainly reflected in the similar biological functions of GSDMD and MLKL, both of which mediate cell membrane perforation, but the mechanisms of activation of the two differ significantly. The activation of GSDMD is dependent on the regulation of the inflammasome–caspase‐1/4/5/11 signalling cascade (Shi et al., 2015), whereas the activation of MLKL is achieved through RIPK3‐mediated phosphorylation modification. Of particular interest is the synergistic amplification of these death pathways in systemic inflammatory states, such as sepsis. DAMPs released by necroptosis–apoptosis can activate inflammasomes, which can trigger pyroptosis by activating the NLRP3 inflammasome to promote the cleavage of GSDMD (Pandey et al., 2024), resulting in the formation of a necroptotic–apoptotic–pyroptotic cascade amplification loop, which can promote the inflammatory damage of tissues synergistically. These findings provide an important theoretical basis for the in‐depth analysis of the molecular mechanisms of disease development and the development of new intervention strategies.

## 3 PATHOLOGICAL ROLE OF RIPK1/RIPK3/MLKL IN SEPSIS‐ASSOCIATED ACUTE KIDNEY INJURY

### 3.1 Direct tubular injury

**FIGURE 1:**
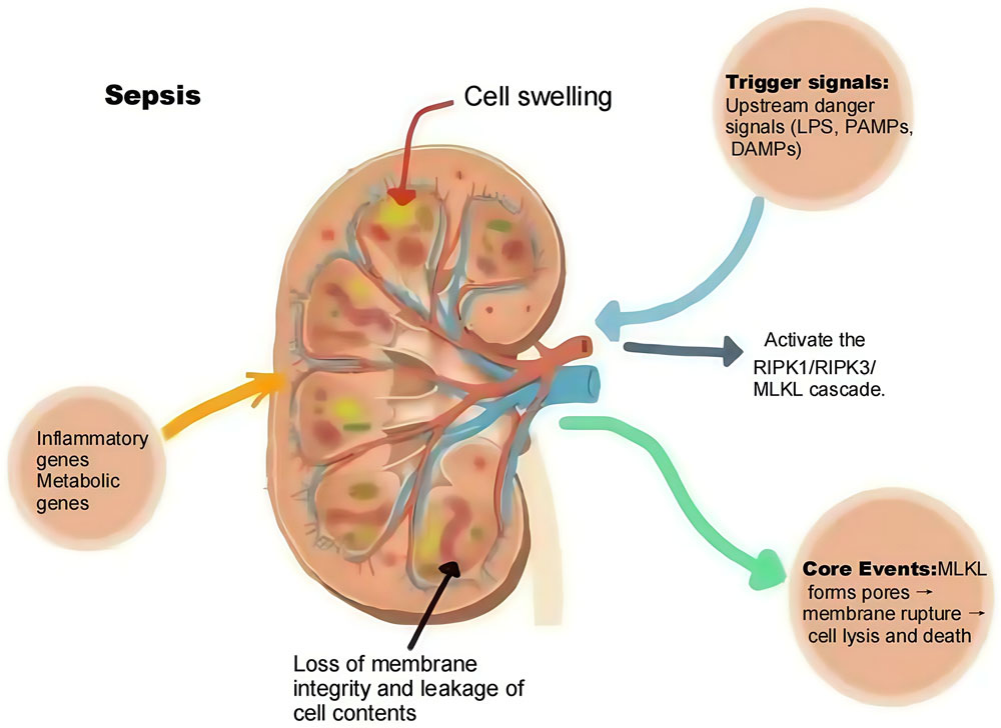
Focuses on the mechanism of kidney injury caused by sepsis. Upstream danger signals(such as LPS, PAMPs, DAMPs) act as trigger signals to activate the RIPK1/RIPK3/MLKL cascade. The core event is that MLKL forms pores, causing cell membrane rupture, which in turn leads to cell lysis and death. During this process, cell swelling, loss of cell membrane integrity and leakage of cell contents also occur. At the same time, inflammatory genes and metabolic genes are also involved, jointly promoting the occurrence and development of kidney injury. Legend: Lipopolysaccharide (LPS), Pathogen ‐ Associated Molecular Patterns (PAMPs), Damage ‐ Associated Molecular Patterns (DAMPs), Receptor ‐ Interacting Serine/Threonine ‐ Protein Kinase (RIPK), Mixed Lineage Kinase Domain ‐ Like Protein (MLKL).

In the complex pathology of SA‐AKI, necroptosis–apoptosis of renal tubular epithelial cells is one of the key mechanisms leading to renal dysfunction. Studies have shown that activation of the RIPK1/RIPK3/MLKL pathway triggers renal tubular epithelial cells to undergo a characteristic programmed necrotic process. This pathological change has typical morphological features, which are manifested by a significant increase in cell volume, loss of plasma membrane integrity and spillage of cytoplasmic contents (Zhou et al., 2024) (Figure 1). Electron microscopic observations showed that the activated MLKL protein oligomerizes and specifically localizes to the surface of the lysosomal membrane, and this altered localization triggers an increase in lysosomal membrane permeability, which, in turn, activates the lysosome‐dependent cell death pathway (Liu et al., 2023). Indirectly, the effect of MLKL oligomerization on the structure and function of the cell membrane is illustrated, which is compatible with the idea that the insertion of MLKL oligomerization into the cell membrane to form a pore leads to cell lysis. The importance of this mechanism was verified by pathohistological examination, in which typical signs of tubular injury were observed in renal biopsy specimens from patients with SA‐AKI, including large epithelial cell detachment, tubular lumen protein involved in tubular formation, and progressively worsening interstitial oedema (Li et al., 2024). These structural changes were correlated significantly with the degree of deterioration in renal function indices, providing direct clinicopathological evidence for the crucial role of necroptosis–apoptosis in the pathogenesis of SA‐AKI.

### 3.2 Microcirculatory disorders and endothelial damage

Necroptosis–apoptosis not only affects renal tubules, but also exacerbates the pathological process of SA‐AKI through the death of vascular endothelial cells. Studies in animal models revealed that renal microvascular endothelial cells in the septic state highly expressed RIPK3 and MLKL, which triggered necroptosis–apoptosis, leading to microthrombosis and increased capillary permeability (Wu et al., 2024) (Figure 1). This process further reduces renal perfusion and triggers local ischaemia and oxidative stress. These changes lead to a further reduction in local renal perfusion, which, in turn, triggers local ischaemia and oxidative stress. In addition to this, a significant reduction in microvessel density was observed in the cortical region of the kidney in AKI (Prommer et al., 2018). Therefore, it can be hypothesized that endothelial cell injury is an important driver of microcirculatory impairment in SA‐AKI, and its destructive effect on the renal microcirculation plays an indispensable role in disease progression.

### 3.3 Inflammatory amplification and immune dysregulation

Necroptosis–apoptosis exacerbates the inflammatory response in pathological conditions through multiple pathways, and its central mechanism involves the release of DAMPs. When necroptosis–apoptosis occurs, the intracellular stores of HMGB1, ATP and other DAMPs spill over to the extracellular milieu in large quantities, which, in turn, activates a cascade of responses in the natural immune system. DAMPs can contribute significantly to the polarization of macrophages towards a pro‐inflammatory M1 phenotype by binding to the receptor for advanced glycosylation end products on their surface and inducing the formation by neutrophils of Neutrophil Extracellular Traps (NETs), and these NETs cause secondary damage to vascular endothelial cells and parenchymal tissues while clearing pathogens (Komai et al., 2017). In addition, there is a self‐reinforcing vicious cycle between necroptosis–apoptosis and the cytokine storm. In pathological conditions, pro‐inflammatory factors, such as TNF‐α and IL‐1β, secreted by macrophages and T cells can promote the formation of the RIPK1/RIPK3 complex through activation of TNFR1, which, in turn, phosphorylates MLKL proteins and induces perforation of the cell membrane. The newly occurring necroptosis–apoptosis, in turn, further releases DAMPs and cellular inclusions, forming a positive feedback regulatory loop (Zhou et al., 2024). Clinical data demonstrated that IL‐1β and TNF‐α levels in renal tissues from SA‐AKI patients were significantly correlated with necrotic apoptotic markers, such as phosphorylated MLKL (Liu et al., 2024), suggesting that targeting this pathway might alleviate excessive inflammatory responses, expanding the understanding of the role of necroptosis–apoptosis in chronic inflammatory diseases.

## 4 EVIDENCE FROM EXPERIMENTAL AND CLINICAL STUDIES

### 4.1 Studies in animal models

In recent years, experimental studies based on mammalian models have provided important evidence to elucidate the mechanism of necroptosis–apoptosis in SA‐AKI. Utilizing a pair of traditional modeling techniques, i.e., caecum ligation perforation (CLP) and LPS induction, the researchers systematically assessed the functions of key regulators of programmed cell death. Particularly noteworthy is that when experimental animals were deficient in *RIPK3* or *MLKL* genes, their renal tissues showed a significant protective effect in the septic state. This protective effect was mainly reflected in three aspects: (1) the concentration of Scr, an important clinical index reflecting renal function, was significantly reduced; Moreover, there was a notable decrease in necrosis among the renal tubular epithelial cells; and (3) the renal injury scores were significantly improved, as seen by histopathological tests such as Haematoxylin and Eosin staining (Pefanis et al., 2023).

In the study of intervention strategies, specific small molecule inhibitors show potential therapeutic value. For example, GSK872, a highly selective inhibitor of RIPK3, can effectively block the activation of the necroptosis–apoptosis signalling pathway, whereas necrosulphonamide inhibits the cell membrane perforation function of MLKL by targeting its oligomerization process (Adameova et al., 2022; Bai et al., 2023). However, the translation of these experimental therapeutic agents to the clinic still has multiple obstacles: (1) the selectivity of existing compounds to their targets needs to be optimized to avoid interfering with other important signalling pathways; (2) the possible hepatorenal toxicity of long‐term use of the drugs requires a more comprehensive safety assessment; and (3) the complex pathophysiological changes in sepsis patients, such as the presence of systemic inflammatory storms and microcirculatory disorders, might significantly affect the therapeutic effects of the drugs. These findings suggest that future studies need to develop more specific intervention strategies.

### 4.2 Clinical relevance

Clinicopathological studies have provided important clues to the pathogenesis of SA‐AKI. Systematic analysis of renal tissues from human SA‐AKI patients revealed that the expression levels of RIPK1, RIPK3 and MLKL, key mediators of regulatory cell death, showed a significant upregulation. Immunohistochemical localization studies revealed that the distribution of these pro‐necrotic apoptotic proteins in the renal units was cell specific, mainly concentrated in the cytoplasmic segments of proximal tubular epithelial cells and the basement membrane side of glomerular capillary endothelial cells, and this pattern of distribution was highly compatible with the experimental results of the rodent SA‐AKI model (Sureshbabu et al., 2018). In addition, the activation status of necroptosis–apoptosis during the disease process can be assessed quantitatively by detecting the phosphorylated form of MLKL (p‐MLKL) in circulating blood, suggesting that it might serve as an early diagnostic or prognostic assessment of SA‐AKI (Jin et al., 2024). Based on these findings, dynamic monitoring of p‐MLKL is expected to be incorporated into the early warning system for SA‐AKI. However, large‐scale clinical studies are needed to validate the specificity of these markers and their value in dynamic monitoring.

## 5 THERAPEUTIC TARGETS AND TRANSLATIONAL MEDICINE

### 5.1 Current status of development of existing inhibitors

In the therapeutic field of SA‐AKI, important breakthroughs have been made in the development of drugs targeted against key regulators of necroptotic–apoptotic signalling pathways. Studies have shown that RIPK1, RIPK3 and MLKL, as core regulators of programmed cell death, have become important targets for drug development. Currently, several small molecule inhibitors have shown good therapeutic effects in animal models. And small molecule inhibitors targeting key molecules of necroptosis–apoptosis (RIPK1, RIPK3 and MLKL) have shown potential in therapeutic studies of SA‐AKI.

The RIPK1/RIPK3/MLKL pathway exhibits significant heterogeneity across different types of renal injury models. In sepsis‐related models, pathogen recognition mechanisms differ importantly: Gram‐negative bacteria act primarily through the Toll‐like receptor 4/MyD88/RIPK3 axis, contributing to ∼75% of renal tubular injury; in contrast, Gram‐positive bacteria rely more on the NOD2/RIPK2‐MLKL pathway (Fu et al., 2024). This difference suggests that pathogen‐specific therapeutic strategies might be necessary.

In terms of temporal dynamics, pathway activation shows distinct phasic characteristics. In the early phase (0–6 h), LPS recognition triggers RIPK1 phosphorylation; subsequently (6–12 h), necrosomes form; at 12–24 h, MLKL translocation and disruption of membrane integrity occur; and finally (24–72 h), inflammatory amplification is induced through DAMP release. This temporal profile provides important guidance for selecting the timing of intervention.

Comparing pharmacodynamic data across models reveals that in the CLP model, the pathway contributes 68% ± 5% to injury, and GSK872 reduces Scr by 39% and improves 28‐day survival by 22%; in the LPS model, the pathway contributes 54% ± 7%, with a 51% reduction in IL‐6 but no significant effect on survival; and in the ischaemia–reperfusion model, the pathway contributes 42% ± 6%, mainly manifesting as a two‐grade improvement in histology. These differences highlight the need to optimize treatment regimens based on model characteristics.

In terms of RIPK3 inhibitors, GSK872 and RIPA‐56 showed significant nephroprotective effects in animal models. For example, in LPS‐ or CLP‐induced septic mice, GSK872 was able to lower serum creatinine levels, reduce tubular necrosis and improve microcirculatory impairment (Xue et al., 2022). RIPA‐56, in contrast, attenuates systemic inflammatory responses and organ damage by selectively inhibiting the kinase activity of RIPK3 (Chen et al., 2022). However, the clinical translation of these inhibitors faces challenges. For instance, the metabolic kinetic characteristics of the drugs show that the half‐life of the available compounds in vivo is generally short, and there is a significant first‐pass effect; tissue distribution studies show that the accumulated concentration of the drugs in the kidney often fails to reach the effective therapeutic dose, whereas abnormal accumulation occurs in non‐target organs, such as the liver; and in vitro experiments have found that some of the inhibitors at high concentrations might interfere with other kinase signalling pathways, producing unintended pharmacological effects. These factors have seriously restricted the clinical application of the drugs concerned. The development of inhibitors of MLKL is relatively lagging, mainly owing to the fact that MLKL is not a kinase but disrupts cell membrane integrity through oligomerization (Hildebrand et al., 2014), making it difficult for traditional small molecule drugs to block its function directly.

The current scientific research work is mainly being carried out in two directions: (1) focusing on the research and development of MLKL conformation inhibitors, through the design of specific compounds, such as necrosulphonamide derivatives, effectively interfering with the localization process of the MLKL protein in the cell membrane; and (2) we aim to improve the drug delivery system by using advanced technology, such as nanoparticle carriers and liposome encapsulation, in order to enhance the specific accumulation capacity of the drug in the renal tissues, while minimizing the toxic side effects on other parts of the body. These two research directions are complementary to each other, and together they are driving breakthroughs in the field of MLKL‐targeted therapy. Although promising, preclinical data on MLKL inhibitors are limited, and further validation of their safety and long‐term efficacy is needed.

### 5.2 Progress in targeted drug development

Current drug development targeting this pathway focuses on three directions, which include as follows: Research and development of RIPK1 allosteric inhibitors, research and development of bifunctional modulators and prodrug design. Among RIPK1 allosteric inhibitors, DNL758 has completed its first human trial (NCT04211693), with good tolerance at 50 mg twice daily, but only a 28% reduction in urinary RIPK1 (Gong et al., 2019). Newer compounds, such as R552, are optimized for peripheral organs by reducing brain permeability (10‐fold reduction) (Chaouhan et al., 2022). Studies on the cryo‐electron microscopy structure of RIPK3 provide a high‐precision structural basis for the design of allosteric inhibitors, facilitating the development of new‐generation compounds (Su H, et al., 2025).

Dual‐function modulators, represented by ADU‐S100, simultaneously block RIPK1 and stimulator of interferon genes (STING) pathways, showing synergistic effects in a sepsis‐induced acute respiratory distress syndrome (ARDS) model (Wang et al., 2011). In prodrug design, pro‐non‐steroidal anti‐inflammatory drugs (Pro‐NSA) is specifically activated in proximal tubules via SGLT2‐mediated uptake, enhancing targeting (Lawlor et al., 2015).

Considerable advancements have occurred in the field of biological treatments. Anti‐MLKL nanobodies exert inhibitory effects by binding to the 4HB domain (*K*_D_ = 2.3 nM) (Qian et al., 2021). The latest preclinical data show that this nanobody can reduce renal injury scores by 60% in non‐human primate models (Zhao et al., 2021). Engineered exosomes modified with CD133 aptamers show significantly increased renal enrichment to 21% (Wu et al., 2021). These advancements offer novel choices for targeted treatment.

### 5.3 Combination therapy strategies

Given that the pathological mechanism of SA‐AKI involves necroptosis–apoptosis, inflammatory storm and microcirculatory disorders, single‐targeted therapies might have limited effects. Therefore, in recent years, researchers have begun to explore multi‐target synergistic intervention strategies to improve clinical efficacy and reduce the risk of drug resistance.

Anti‐necroptosis–apoptosis and anti‐inflammatory drugs can be combined. For example, the combination of an RIPK3 inhibitor (e.g. GSK872) with an IL‐6 receptor antagonist (e.g. tolizumab) showed a synergistic effect in a sepsis model: the former effectively reduced programmed necrosis of renal tubular epithelial cells, and the latter inhibited inflammatory amplification, leading to a more comprehensive improvement in renal function (Sureshbabu et al., 2018). This dual intervention strategy not only reduced renal tissue damage but also improved glomerular filtration rate. Likewise, the combination of MLKL inhibitors with TNF‐α blockers (e.g. etanercept) further reduced cytokine storm injury (Chaouhan et al., 2022). This combination therapy reduces serum levels of pro‐inflammatory factors, such as TNF‐α and IL‐1β, in addition to reducing oligomerization of MLKL in renal tissues, resulting in a more comprehensive protection of renal function.

Drug treatments can be combined with blood purification techniques. For instance, continuous renal replacement therapy removes inflammatory mediators (e.g. IL‐1β, HMGB1) and necroptosis–apoptosis‐associated molecules (e.g. free MLKL) from the bloodstream (Fang & Jiang, 2016), whereas the drugs (e.g. RIPK1 inhibitors) block the cell‐death signalling at source. This joint strategy is more effective in improving survival and renal function in septic animals.

**FIGURE 2:**
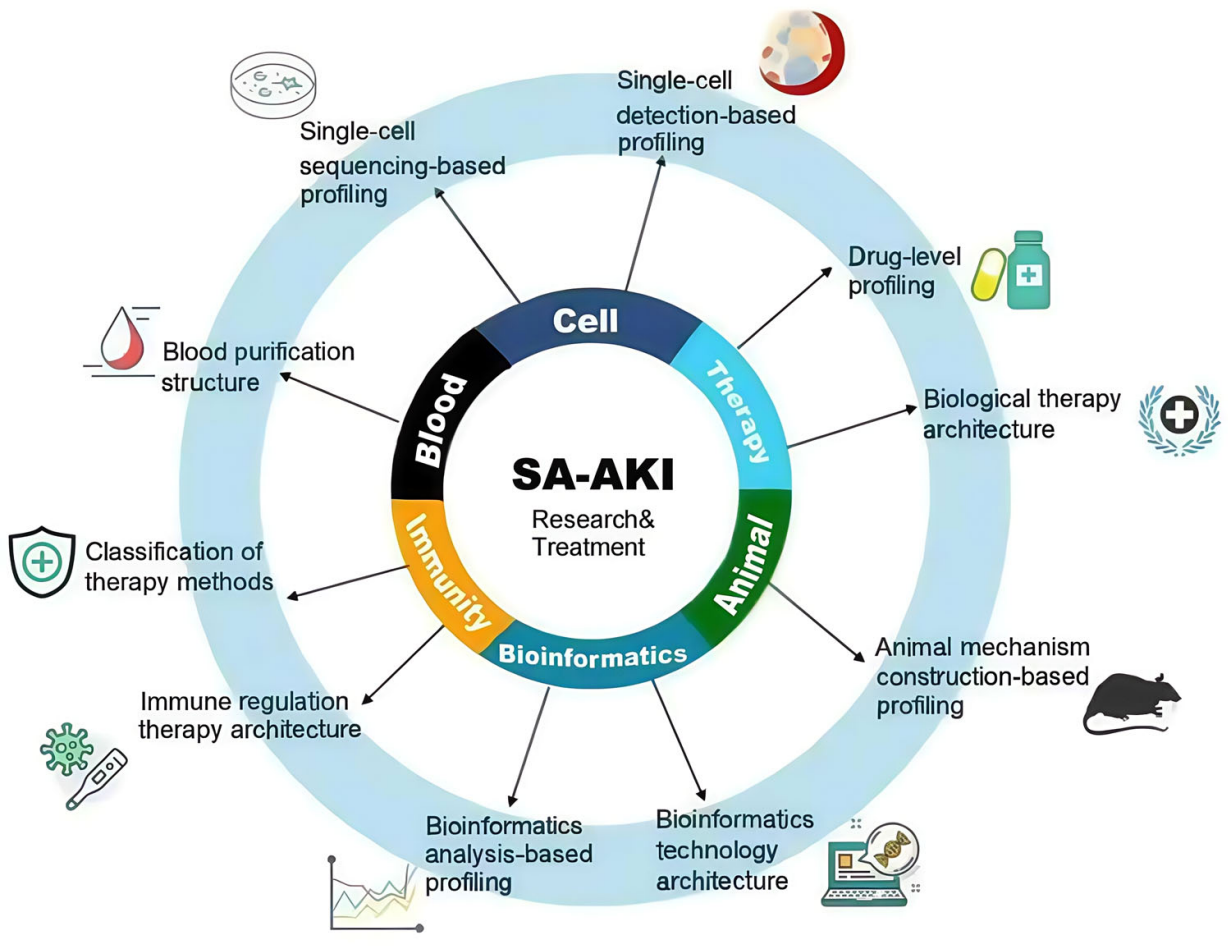
Shows the multi‐dimensional research and diagnosis‐treatment framework of SA‐AKI. At the cellular level, there are analyses based on single‐cell sequencing and single‐cell detection; at the treatment level, it covers drug‐level analysis and biological therapy architecture;at the animal level, it involves analysis based on animal mechanism construction;at the bioinformatics level, it includes bioinformatics analysis and technical architecture;at the immune level, there are immune regulation therapy architecture and classification of treatment methords; in addition, there are contents related to blood purification structure, which provide support for the research, diagnosis and treatment of SA‐AKI from multiple dimensions.

In the future, with the in‐depth understanding of the molecular mechanism of SA‐AKI, the combination therapy strategy is expected to develop in the direction of more precision and individualization, including the optimization of a dynamic treatment regimen based on biomarkers, in addition to application of the combination of new targeted drugs and an artificial intelligence‐assisted blood purification system (Figure 2).

### 5.4 Challenges and safety

Although targeting the necroptotic–apoptotic pathway provides a new intervention strategy for the treatment of SA‐AKI, its potential clinical safety issues need to be evaluated cautiously. The primary challenge is the potential impact of systemic inhibition of RIPK1/RIPK3/MLKL on immune defence. These molecules play important roles in anti‐infection immunity; for example, RIPK3 is involved in antiviral responses (Downey et al., 2017), and MLKL deficiency might impair bacterial clearance. Animal studies have shown that RIPK3‐deficient mice have an increased bacterial burden in the early stages of infection (Stutz et al., 2018). Therefore, the balance between the nephroprotective effects of anti‐necroptosis–apoptosis therapy and potential immunosuppressive side effects must be weighed carefully during clinical translation. In addition, the lack of organ targeting of drug delivery systems is an important factor limiting their clinical application. Existing inhibitors can affect other organs with high RIPK3 expression, such as the heart and intestine, leading to off‐target toxicity. Thus, creating kidney‐specific delivery methods, for instance, drug delivery systems utilizing nanotechnology, is essential (Kuskov et al., 2025). Finally, disease heterogeneity in SA‐AKI patients places greater demands on the development of individualized treatment plans. SA‐AKI patients are heterogeneous in terms of aetiology and course of disease, and in the future, biomarker stratification might be required for precise selection of the inhibitor type and course of therapy. Necroptosis–apoptosis inhibitors have a promising future in SA‐AKI therapy, and although the field still faces many challenges, necroptosis–apoptosis‐targeted therapies are expected to become an important part of precision medicine practice in SA‐AKI through multidisciplinary collaboration to optimize drug design, improve delivery systems and carry out rigorous preclinical safety evaluations.

## 6 FUTURE RESEARCH DIRECTIONS

The heterogeneity of SA‐AKI is a key factor influencing clinical treatment response and prognosis. Future studies should focus on molecular typing based on necrotic–apoptotic activity to distinguish subgroups of SA‐AKI with different pathophysiological features. Through single‐cell RNA sequencing (Schumacher et al., 2021) or spatial transcriptome technology (Noel et al., 2022), the expression profiles of necroptosis–apoptosis marker genes, such as *RIPK3* and *MLKL*, can be characterized in renal tissues, and predictive models can be established in combination with clinical parameters. This precise typing will facilitate the development of individualized treatment strategies and provide the basis for enrolment criteria in clinical trials. In addition to this, the role of necroptosis–apoptosis in SA‐AKI might exacerbate renal tubular injury through metabolic reprogramming. Future integration of transcriptomic, proteomic and metabolomic data is needed to resolve systematically the interaction network of the RIPK1/RIPK3/MLKL pathway with key molecules of metabolism (Cavill et al., 2015). In addition, multi‐omics validation of organoid models or patient‐derived samples might reveal targets conserved across species and provide theoretical support for combined interventions (Rodboon et al., 2022; Wang et al., 2024). In summary, future research needs to integrate clinical typing, multi‐omics mechanism analysis and technological innovation to form a complete research chain from basic to translational and, ultimately, improve the diagnostic and therapeutic dilemma of SA‐AKI.

## 7 CONCLUDING REMARKS

The pathophysiological process of SA‐AKI involves the interaction of multiple molecular mechanisms, among which the programmed necrosis signalling pathway mediated by RIPK1/RIPK3/MLKL has been shown to be a key factor driving the irreversible damage to renal tubular epithelial cells. Recent studies have revealed that aberrant activation of this pathway not only directly triggers disruption of cell membrane integrity and release of intracellular contents, but also triggers a systemic inflammatory storm through the release of DAMPs, exacerbating microvascular endothelial dysfunction and localized ischaemia–reperfusion injury, which ultimately leads to multi‐organ failure. In preclinical studies, therapeutic strategies that selectively inhibit RIPK3 or block MLKL oligomerization showed significant renoprotective effects in both CLP‐induced sepsis and renal ischaemia–reperfusion models, and Scr and blood urea nitrogen levels could be reduced, while pathology showed a significant reduction in the area of renal tubular necrosis. These findings provide a solid theoretical basis for the development of new targeted therapies.

However, the translation of laboratory results into clinical applications faces a number of key issues that need to be resolved. First, at the level of disease diagnosis, there is a lack of biomarkers that can accurately distinguish necroptosis–apoptosis‐dominant SA‐AKI, and the existing clinical indicators, although reflecting renal tubular injury, are unable to identify the activation state of programmed necrosis specifically. Second, in terms of drug development, although RIPK3 inhibitors have demonstrated inhibitory potency in in vitro experiments, their pharmacokinetic properties in vivo and their potential off‐target toxicity limit their prospects for clinical application. In addition, sepsis patients exhibit a high degree of clinical heterogeneity, and different pathogenic infections, comorbidities and genetic backgrounds might lead to significant differences in response to targeted therapies, but reliable predictive models that can be used for patient stratification are not yet available.

To break through these translational medicine bottlenecks, future research needs to adopt a multidisciplinary synergistic innovation strategy. In basic research, cutting‐edge technologies, such as single‐cell transcriptomics and spatial metabolomics, should be integrated to analyse systematically the dynamic regulatory network of programmed necrotic signalling in different SA‐AKI subtypes; in drug optimization, highly selective variant inhibitors can be designed with the help of artificial intelligence‐assisted molecular docking technology, or renal tubule‐targeted nano‐delivery systems can be developed to increase the local concentration of the drug; and in the phase of clinical verification, standardized multicentre cohorts need to be established. Successfully translating clinical research involves tackling a number of crucial challenges. For patient stratification, high‐throughput screening identified the rs201062358 single nucleotide polymorphism as significantly associated with treatment response (odds ratio = 3.2) (Petrie et al., 2018). Urinary exosome sequencing can effectively distinguish responders (area under the curve = 0.91), providing a basis for precision therapy (Xia et al., 2016). The 2025 World Health Organization Guidelines for the Treatment of Acute Kidney Injury specifically emphasize the central role of biomarkers in patient stratification, which is highly consistent with the stratification strategy of this study.

The optimization of combined treatment regimens needs to consider systematically the synergistic effects of various links, from pathogen clearance (antibiotics) to immune regulation, to cell death inhibition and organ support, forming a complete treatment chain.

### 7.1 Development pathway for the next 5 years

Based on current research progress, the following development pathway can be outlined:

• 2025–2026: Complete phase II studies of RIPK1 inhibitors (*n* = 220) and establish clinical cut‐off values for p‐MLKL.
• 2027–2028: Launch the first nanobody clinical trial and develop an artificial intelligence‐assisted protocol optimization platform.
• 2029–2030: Integrate personalized treatment guidelines into KDIGO and obtain approval for companion diagnostic kits.

This path matches the disease burden data revealed by the Chinese SA‐AKI multicenter epidemiological study and can prioritize coverage of high‐risk populations (Wang, L. et al. 2024).

This proposed developmental pathway outlines a potential strategy for mitigating the high disease burden associated with SA‐AKI. The intervention priorities are informed by the epidemiological profile of SA‐AKI, as characterized in studies such as the descriptive analysis from Beijing, China (Wang et al., 2021). This evidence‐based approach enables effective prioritization of resource allocation and strategic planning for high‐risk populations.

### 7.2 Evaluation of multi‐dimensional combination therapies

Different combination regimens have distinct characteristics:

• RIPK inhibitor plus IL‐6 antagonists: Block the death–inflammation loop, validated in animal models but might increase the risk of secondary infections.
• MLKL inhibitors + mitochondrial protectants: Simultaneously protect membrane integrity and energy metabolism, supported by in vitro organoid chip data, but metabolic interactions remain unclear.

Selection of these regimens requires efficacy and risk to be balanced, with individualized plans based on patient‐specific conditions. In addition, drug sensitivity testing using patient‐derived renal organoids or humanized mouse models is expected to provide a predictive reference for the selection of individualized treatment regimens.

## AUTHOR CONTRIBUTIONS

Huijun Yin: Writing—review & editing, Writing—original draft, Visualization, Conceptualization. Jingyi Wang: Writing—review & editing, Visualization. Huirong Han: Writing—review & editing, Writing—original draft, Supervision, Software, Resources, Project administration, Funding acquisition, Conceptualization. All authors approved the final version of the manuscript and agree to be accountable for all aspects of the work in ensuring that questions related to the accuracy or integrity of any part of the work are appropriately investigated and resolved. All persons designated as authors qualify for authorship, and all those who qualify for authorship are listed.

## CONFLICT OF INTEREST

None declared.
